# The contribution of sport participation to overall health enhancing physical activity levels in Australia: a population-based study

**DOI:** 10.1186/s12889-015-2156-9

**Published:** 2015-08-20

**Authors:** RM Eime, JT Harvey, MJ Charity, MM Casey, JGZ van Uffelen, WR Payne

**Affiliations:** Institute of Sport, Exercise and Active Living, Victoria University, PO Box 14428, Melbourne, Victoria 8001 Australia; School of Health Sciences and Psychology, Federation University Australia, PO Box 663, Ballarat, Victoria 3353 Australia

## Abstract

**Background:**

The contribution of sport to overall health-enhancing leisure-time physical activity (HELPA) in adults is not well understood. The aim was to examine this in a national sample of Australians aged 15+ years, and to extend this examination to other ostensibly sport-associated activities.

**Methods:**

The 2010 Exercise, Recreation and Sport Survey (ERASS) was conducted by telephone interview in four quarterly waves. Data from this survey were analysed to categorise leisure-time physical activity (LTPA) as HELPA or non-HELPA, and to categorise HELPA activities and sessions of HELPA activity by setting and frequency. The contribution of sport to HELPA was estimated, both directly through activities and settings classified as sport per se, and indirectly through other fitness activities ostensibly related to preparation for sport and enhancement of sport performance.

**Results:**

Of 21,602 respondents, 82 % reported some LTPA in the 12 months prior to the survey. In aggregate, respondents reported 37,020 activity types in the previous 12 months, of which 94 % were HELPA. Of HELPA activities, 71 % were non-organised, 11 % were organised but not sport club-based, and 18 % were sport club-based. Of all sport activities, 52 % were HELPA. Of sport HELPA, 33 % was sport club-based and 78 % was undertaken ≥12 times/year. Sport club members were significantly more likely to have participated in running, but significantly less likely to have participated in walking or aerobics/fitness training, than non-club members.

**Conclusions:**

Club sport participation contributes considerably to LTPA at health enhancing levels. Health promotion policies, and more specifically physical activity policies, should emphasize the role of sport in enhancing health. Sport policy should recognise the health-promoting role of community-based sport in addition to the current predominant focus on elite pathways.

## Background

Regular participation in physical activity (PA) is imperative for good health [1]. Health benefits include decreased risks of chronic physical and mental conditions such as diabetes, cardiovascular disease and depression [1, 2]. Public health guidelines for adults stipulate a minimum level of 150 min of moderate intensity PA per week to achieve these health benefits [3]. Activities of at least moderate intensity are often referred to as health-enhancing PA. Research suggests that 31 % of the world’s population is not meeting this minimum PA level for health benefits [4]. This lack of regular PA causes 6-10 % of the burden of disease worldwide and 9 % of premature mortality [2]. Physical inactivity is, therefore, a public health priority.

People can be active in different PA domains, including active transport, domestic, occupational and leisure-time physical activity (LTPA) [5]. A range of studies indicate unique health benefits of LTPA compared to other PA domains in people aged 15 years and older [5–9]. For example, LTPA was associated with better self-reported health and lower obesity rates in European adults, whereas there was no association between total PA level and these outcomes [5]. The benefits of LTPA do not only apply to physical health. For example, a study in adult women found a beneficial association between symptoms of depression and LTPA, but not with occupational PA (including household chores), or active commuting [8]. Another study in three age cohorts of Australian women reported that the positive associations between LTPA and quality of life were attenuated after taking non-LTPA into account [10].

More specifically, sport has been associated with better health-related quality of life (HRQoL) in adults than other forms of LTPA [6, 8, 9], and with lower all-cause mortality compared with non-participation [7]. Specifically, participation in low to moderate amounts of club sport participation has been found to contribute to greater physical health benefits than PA participation in other settings [11]. However, notwithstanding this evidence for additional health benefits of sport over other forms of PA for quality of life and physical health, the mechanisms are not well understood.

It is likely that the social nature of organised sport participation plays a larger role in improving social and mental health, and thus quality of life, than other forms of PA [6, 11]. This view has been supported by a systematic review of the psychological and social health benefits of sport participation [11]. The ‘Health through Sport’ conceptual model presented in this systematic review provides an overview of the specific improved health outcomes of club-based or team-based sport due to the social nature of participation [11].

Whilst there is some evidence of the role that sport can play for different health domains, little is known about the magnitude of the contribution of sport participation to overall health enhancing PA levels in adults. Knowledge of this would be invaluable to inform specific PA interventions. The aims of this study were twofold: 1) to investigate the direct contribution of sport to overall health-enhancing LTPA levels in adults; and ii) to investigate the indirect contribution of sport to LTPA levels through an examination of the association between sport participation and participation in other modes types of LTPA which might be undertaken as preparation or training for club sport.

## Methods

Data from the 2010 Exercise, Recreation and Sport Survey (ERASS) [12] were obtained from the Australian Sports Commission (ASC), which commissioned the survey. The usefulness of the ERASS survey as a national surveillance of habitual PA behaviours has been established [13, 14].

Quarterly survey samples for ERASS were selected from all persons aged 15 years and over living in occupied private dwellings using computer-assisted telephone interviewing. In each quarter during the 2010 calendar year approximately 3400 persons were sampled from all Australian states and territories. Verbal informed consent was indicated by the respondents’ willingness to participate in the telephone survey. Ethics approval was granted by the Human Research Ethics Committee of Federation University Australia for the secondary analysis of the ERASS data.

After explaining the purpose and format of the ERASS questionnaire, interviewers asked respondents if they had participated in any LTPA for exercise, recreation or sport in the last 12 months (as opposed to PA associated with employment, housework or garden chores). If the response was ‘yes’, respondents were then asked to report what activities they had participated in during this time period (up to a maximum of 10 activities). Activities were classified, first into 170 categories, then further categorised into 95 categories which we refer to as ‘types’ of activity. Respondents were then requested, for each reported activity type, to indicate whether any of the activity had been organised by a club, association or any other type of organisation. If any of the activity had been organised, a further question then inquired as to what type of club, association or organisation had organised the activity (fitness, leisure or indoor sports centre that required payment for participation; sport or recreation club or association that required payment of membership, fees or registration; work; school; other). From responses to the above questions three dichotomous measures were derived, indicating: (1) whether there was any participation in PA for exercise, recreation or sport in the past 12 months (yes/no); (2) for each type of activity, whether any of the activity was organised (yes/no); and (3) if so, was the activity organised by a sport or recreation club or association that required payment of membership fees or registration (herein referred to as club) (yes/no).

Respondents were also asked how many times (sessions or episodes) they had participated in each of their nominated types of activity during the previous 12 months. After consultation with peak sport governing bodies, a further dichotomous variable was generated in this study for each activity: frequency ≥12 times, notionally representing ‘regular’ participation (at least once per week for a 12 week season or once per month all year round); and frequency <12 times, representing ‘occasional’ participation.

Of the up to ten types of activity nominated, respondents were then asked to nominate up to three ‘top activities’. For each of these, they were asked how many times they had participated during the previous 2 weeks, from which a further dichotomous variable was generated in this study for each activity: frequency ≥2 times, notionally representing ‘regular’ participation (at least once each week); and frequency <2 times, representing a less regular or ‘occasional’ level of participation.

While ERASS provides data about frequency of activities in both the short term (2 weeks) and longer term (12 months), it does not include data about duration or intensity of activity. In this study, each of the 95 ERASS PA types was categorised as either a HELPA or non-HELPA activity, according to the MET value (metabolic equivalent of task) of the activity [15]. A MET value of 3.5 or more was classified as HELPA, in accordance with the specification of Merom et al. [14]. An activity was also classified as sport if it was administered by a state sporting association (SSA) and/or national sporting organisation (NSO) recognised as such by the Australian Sports Commission [16]. Both HELPA PA types generally and HELPA sports were then categorised according to level of organisation of participation (sports club-based/other organised/non-organised).

Reported LTPA, in both the previous 12 months (for up to 10 activities) and the previous 2 weeks (for up to three ‘top activities’), was quantified in two ways: in terms of the number of types of activity reported and in terms of the number of sessions reported for each type. Aggregated counts of activity types and sessions were apportioned into the various categories (HELPA/non-HELPA; sport/non-sport; level of organisation; regular/occasional) and percentage breakdowns were calculated.

In order to investigate the possibility of additional indirect contributions of club sport to HELPA, analysis was also undertaken of the association between playing club sport and participation in four selected types of activity considered by the researchers to be potentially beneficial to club sport: aerobics/fitness training, running, weight training and walking. For each of these four types of activity, those who participated in the selected types of activity and also in any sport at club level, were compared to the total number of people participating in the particular types of activity and to the total number of people participating in any sport at club level. These analyses were conducted for all participants, and also separately within categories of gender, age and geographical region.

Technically, for each of the four types of activity, three counts were extracted from a 2 × 2 cross-tabulation of club sport participation v participation in the particular type of activity. The three counts were: the number who participated in the particular type of activity (column a in Tables 3 and 4), the number who participated in sport at the club level (column b, which is constant across all four types of activity), and the number who did both (column c). The number who did both was then expressed as a percentage of those who participated in the particular type activity (column d), and of those who participated in club sport (column e). Column d indicates the percentage of those who undertook the particular PA who also played club sport. Conversely, column e indicates the percentage of those who played club sport who also undertook the particular PA. Additionally, for each cross-tabulation a measure of concordance (the gamma statistic) was calculated, indicating the extent to which participation in club sport was associated with participation in the particular activity. Like a correlation coefficient, gamma can take values between −1 and +1. Positive values of gamma indicate that club sport participants were more likely than non-club sport participants to participate in the particular activity, and negative values of gamma indicate that sport participants were less likely than non-sport participants to participate in the particular activity.

All analyses used ERASS data weighted at the state, region (metropolitan, non-metropolitan), age-group and gender levels. Population estimates are Australian Bureau of Statistics (ABS) projections for persons in occupied private dwellings at 30 Jun 2010. Analyses were conducted using SPSS Version 19.

## Results

Table 1 provides a breakdown of the survey respondent demographics of gender, age and residential location.

**Table 1:** Survey respondent demographics

Characteristic^a^	Participated in any LTPA in the past 12 months (%)		Did not participate in any LTPA in the past 12 months (%)	
Gender				
Males	8910	(50.1)	1775	(46.3)
Females	8859	(49.9)	2058	(53.7)
Age				
15–29	4524	(25.8)	701	(18.7)
30–49	6678	(38.0)	1103	(29.5)
50+	6349	(36.2)	1940	(51.8)
Region				
Metropolitan	11,658	(65.6)	2405	(62.7)
Non-metropolitan	6111	(34.4)	1429	(37.3)
Total	17,769		3834	

^a^Totals vary slightly due to missing data

Table 2 shows breakdowns of four LTPA metrics: aggregated counts of reported activity types and activity sessions for 12 months prior to the survey and for 2 weeks prior to the survey. In aggregate, people reported 37,020 types of activity and 3,614,593 sessions of activity in the previous 12 months and 26,834 types of activity and 116,573 sessions of activity in the previous 2 weeks.

**Table 2:** Participation in physical activity in the previous 2 weeks and in the previous 12 months

	Types of activity^a^				Sessions^b^			
Physical activity participation: breakdowns	Previous 2 weeks		Previous 12 months		Previous 2 weeks		Previous 12 months	
	n	%	n	%	n	%	n	%
*All activities: by HELPA category*	*26,834*		*37,020*		*116,573*		*3,614,593*	
HELPA (78 types)	25,263	94.1	34,790	94.0	112,149	96.2	3,480,146	96.3
Non-HELPA (17 types)	1572	5.9	2230	6.0	4423	3.8	134,447	3.7
*HELPA activities: by context*	*25,263*		*34,790*		*112,149*		*3,480,146*	
Non-organised	18,348	72.6	24,830	71.4	88,821	79.2	2,672,294	76.8
Organised non-club	2650	10.5	3717	10.7	9012	8.0	307,259	8.8
Club	4265	16.9	6243	17.9	14,316	12.8	500,593	14.4
*HELPA activities: by frequency*	*−*	*−*	*34,662*		*−*	*−*	*2,861,302*	
Occasional (<12 times)	−	−	5253	15.2	−	−	29,605	1.0
Regular (≥12 times)	−	−	29,409	84.8	−	−	2,831,698	99.0
	*21,559*		*−*	*−*	*112,166*		*−*	*−*
Occasional (<2 times)	3208	14.9	−	−	3208	2.9	−	−
Regular (≥2 times	18,351	85.1	−	−	108,958	97.1	−	−
*HELPA activities: by sport category*	*25,263*		*34,790*		*112,149*		*3,480,146*	
HELPA sport (50 types)	11,732	46.4	17,991	51.7	32,790	29.2	1,120,835	32.2
HELPA non-sport (28 types)	13,531	53.6	16,799	48.3	79,360	70.8	2,359,311	67.8
*HELPA sport activities: by context*	*11,732*		*17,991*		*32,790*		*1,120,835*	
Non-organised sport	6313	53.8	10,052	55.9	16,367	49.9	535,857	47.8
Organised non-club sport	1419	12.1	2063	11.5	3235	9.9	118,392	10.6
Club sport	4000	34.1	5876	32.7	13,188	40.2	466,585	41.6
*HELPA sport activities: by frequency*	*−*	*−*	*17,928*		*−*	*−*	*1,121,440*	
Occasional (<12 times)	−	−	3907	21.8	−	−	22,192	2.0
Regular (≥12 times)	−	−	14,021	78.2	−	−	1,099,248	98.0
	*8910*				*32,806*			
Occasional (<2 times)	2011	22.6	−	−	2010	6.1	−	−
Regular (≥2 times	6899	77.4	−	−	30,796	93.9	−	−

Italic is major heading. Non-italic is minor heading

^a^Aggregated counts of types of activity reported by respondents

^b^Aggregated counts of sessions reported by respondents

Table 2 shows breakdowns of: all reported activities and sessions by HELPA category; HELPA activities by context, frequency and sport category; and HELPA sport activities by context and frequency. For all four metrics, well over 90 % of LTPA was classified as HELPA. The breakdowns of the contexts of HELPA activities show that the majority of HELPA activities were undertaken in non-organised contexts, followed by club settings then organised non-club settings. The breakdowns of the frequencies of nominated HELPA activities show that the great majority of HELPA activities were undertaken regularly rather than occasionally.

Around half of the nominated HELPA activities were classified as sport, but sport accounted for a lower proportion (around 30 %) of HELPA sessions. Around half of all nominated HELPA sport activities and sessions were undertaken in organised contexts, and around one third of HELPA sport activities and 40 % of sessions were club-based. The breakdowns of the frequencies of nominated HELPA sport activities show that the great majority of HELPA sport activities were undertaken regularly rather than occasionally. As for HELPA sessions generally, the proportion of HELPA sport sessions undertaken in regular participation was higher than the proportion of HELPA sport activities participated in regularly.

Tables 3 and 4 show the association between playing club sport and participation in four selected physical activities which might be associated with club sport participation through training for the sport: aerobics/fitness training, running, weight training and walking. Table 3 is based on responses regarding the 12 months prior to the survey, and Table 4 is based on responses regarding the 2 weeks prior to the survey. Both tables show results for all ERASS respondents who reported any participation, together with breakdowns by gender, region and age. Walking was reported by the highest number of participants (*n* = 7750 in the previous 12 months), followed by aerobics/fitness training (*n* = 5080), running (2291) and weight training (*n* = 636).

**Table 3:** Participation in selected sport-related physical activity types in the previous 12 months

	a	b	c	d	e	f	g	h	i
Group/ PA type	Number of participants in ‘PA type’	Number of participants in any type of club sport	Number of participants in both PA type and any type of club sport	Percentage of ‘PA type’ participants who also participate in club sport	Percentage of club sport participants who also participate in ‘PA type’	Concordance between participation in ‘PA type’ and club sport	*p*-value	Percentage of non-club sport participants who also participate in ‘PA type’	Difference (percentage points)
				= c/a (%)	= c/b (%)	(Gamma)		(%)	= e-h
All participants									
Aerobics/fitness training	5080	4710	964	19.0	20.5	−0.112	<0.001	24.4	−3.9
Running	2291	4710	724	31.6	15.4	0.280	<0.001	9.3	6.1
Weight training	636	4710	133	20.9	2.8	−0.027	0.575	3.0	−0.2
Walking	7750	4710	1018	13.1	21.6	−0.412	<0.001	39.8	−18.2
Males									
Aerobics/fitness training	1999	2948	502	25.1	17.0	−0.078	0.005	19.3	−2.3
Running	1372	2948	480	35.0	16.3	0.198	<0.001	11.5	4.8
Weight training	413	2948	99	24.0	3.4	−0.096	0.086	4.1	−0.7
Walking	2799	2948	432	15.4	14.7	−0.439	<0.001	30.6	−15.9
Females									
Aerobics/fitness training	3081	1762	462	15.0	26.2	−0.060	0.038	28.6	−2.4
Running	919	1762	244	26.6	13.8	0.338	<0.001	7.3	6.5
Weight training	223	1762	34	15.2	1.9	−0.037	0.686	2.0	−0.1
Walking	4950	1762	586	11.8	33.3	−0.293	<0.001	47.7	−14.4
Aged 15–29									
Aerobics/fitness training	1420	2052	456	32.1	22.2	−0.209	<0.001	30.4	−8.2
Running	834	2052	331	39.7	16.1	0.011	0.785	15.8	0.3
Weight training	204	2052	57	28.0	2.8	−0.259	<0.001	4.7	−1.9
Walking	740	2052	146	19.7	7.1	−0.501	<0.001	18.7	−11.6
Aged 30-49									
Aerobics/fitness training	2055	1597	312	15.2	19.5	−0.236	<0.001	28.1	−8.6
Running	1125	1597	315	28.0	19.7	0.239	<0.001	13.1	6.6
Weight training	269	1597	54	20.1	3.4	−0.015	0.848	3.5	−0.1
Walking	2853	1597	407	14.3	25.5	−0.314	<0.001	39.6	−14.1
Aged 50+									
Aerobics/fitness training	1552	1026	186	12.0	18.1	−0.022	0.599	18.8	−0.7
Running	313	1026	72	23.0	7.0	0.375	<0.001	3.3	3.7
Weight training	156	1026	22	14.1	2.1	0.072	0.554	1.8	0.3
Walking	4015	1026	448	11.2	43.7	−0.110	0.001	49.2	−5.5
Metropolitan									
Aerobics/fitness training	3634	2844	653	18.0	23.0	−0.097	<0.001	26.6	−3.6
Running	1656	2844	500	30.2	17.6	0.300	<0.001	10.3	7.3
Weight training	422	2844	93	22.0	3.3	0.056	0.359	3.0	0.3
Walking	4999	2844	569	11.4	20.0	−0.445	<0.001	39.5	−19.5
Non-metropolitan									
Aerobics/fitness training	1446	1866	312	21.6	16.7	−0.109	0.001	20.0	−3.3
Running	634	1866	223	35.2	11.9	0.270	<0.001	7.2	4.7
Weight training	213	1866	40	18.7	2.1	−0.182	0.023	3.0	−0.9
Walking	2751	1866	448	16.3	24.0	−0.368	<0.001	40.6	−16.6

**Table 4:** Participation in selected sport-related physical activity types in the previous 2 weeks

	a	b	c	d	e	f	g	h	i
Group/ PA type	Number of participants in ‘PA type’	Number of participants in any type of club sport	Number of participants in both PA type and any type of club sport	Percentage of ‘PA type’ participants who also participate in club sport	Percentage of club sport participants who also participate in ‘PA type’	Concordance between participation in ‘PA type’ and club sport	*p*-value	Percentage of non-club sport participants who also participate in ‘PA type’	Difference
				= c/a (%)	= c/b (%)	(Gamma)		(%)	= e-h
All participants									
Aerobics/fitness training	3687	3087	450	12.2	14.6	−0.108	<0.001	17.5	−2.9
Running	1573	3087	319	20.3	10.3	0.227	<0.001	6.7	3.6
Weight training	488	3087	73	15.0	2.4	0.029	0.660	2.3	0.1
Walking	6718	3087	525	7.8	17.0	−0.421	<0.001	33.4	−16.4
Males									
Aerobics/fitness training	1518	1997	245	16.1	12.3	−0.102	0.004	14.7	−2.4
Running	943	1997	197	20.9	9.9	0.076	0.081	8.6	1.3
Weight training	349	1997	62	17.8	3.1	−0.032	0.643	3.3	−0.2
Walking	2453	1997	237	9.7	11.9	−0.435	<0.001	25.5	−13.6
Females								0.0	
Aerobics/fitness training	2169	1089	204	9.4	18.7	−0.040	0.317	20.0	−1.3
Running	629	1089	122	19.4	11.2	0.396	<0.001	5.2	6.0
Weight training	139	1089	12	8.6	1.1	−0.081	0.569	1.3	−0.2
Walking	4265	1089	288	6.8	26.4	−0.308	<0.001	40.4	−14.0
Aged 15–29									
Aerobics/fitness training	1023	1354	229	22.4	16.9	−0.119	0.003	20.5	−3.6
Running	599	1354	152	25.4	11.2	−0.016	0.748	11.5	−0.3
Weight training	159	1354	30	18.9	2.2	−0.207	0.024	3.3	−1.1
Walking	614	1354	58	9.4	4.3	−0.579	<0.001	14.4	−10.1
Aged 30–49									
Aerobics/fitness training	1425	970	108	7.6	11.1	−0.313	<0.001	19.3	−8.2
Running	725	970	128	17.7	13.2	0.226	<0.001	8.8	4.4
Weight training	205	970	32	15.6	3.3	0.131	0.218	2.6	0.7
Walking	2430	970	162	6.7	16.7	−0.427	<0.001	33.3	−16.6
Aged 50+									
Aerobics/fitness training	1200	745	110	9.2	14.8	0.013	0.815	14.5	0.3
Running	234	745	38	16.2	5.1	0.337	0.003	2.6	2.5
Weight training	118	745	12	10.2	1.6	0.069	0.669	1.4	0.2
Walking	3541	745	297	8.4	39.9	−0.064	0.097	43.0	−3.1
Metropolitan									
Aerobics/fitness training	2643	1923	334	12.6	17.4	−0.055	0.077	19.1	−1.7
Running	1159	1923	248	21.4	12.9	0.291	<0.001	7.5	5.4
Weight training	316	1923	58	18.3	3.0	0.178	0.030	2.1	0.9
Walking	4359	1923	302	6.9	15.7	−0.459	<0.001	33.4	−17.7
Non-metropolitan									
Aerobics/fitness training	1044	1163	115	11.0	9.9	−0.217	<0.001	14.6	−4.7
Running	413	1163	71	17.2	6.1	0.069	0.328	5.4	0.7
Weight training	172	1163	15	8.7	1.3	−0.315	0.003	2.5	−1.2
Walking	2359	1163	223	9.5	19.2	−0.359	<0.001	33.5	−14.3

Focusing first on the proportion of club sport participants who engaged in these activities in the previous 12 months (Table 3, column e), overall just over one fifth (20.5 %) of club sport participants had done aerobics/fitness training and a similar proportion (21.6 %) had walked in the 12 months prior to the survey. Around one in six (15.4 %) had run, and a small proportion (2.8 %) had done weight training. Males were more likely than females to run (16.3 % v 13.8 %) or do weight training (3.4 % v 1.9 %), and females were more likely than males to do aerobics/fitness training (26.2 % v 17.0 %) or walk (33.3 % v 14.7 %). The prevalence of aerobics/fitness training diminished slightly as age increased. The proportions of club sport participants engaging in running and weight training each increased a little between the youngest group and the middle-aged group then diminished sharply in the oldest age group. The prevalence of walking increased steadily with increasing age among club sport participants. Aerobics/fitness training, running and weight training were more prevalent among club sport participants in the metropolitan region, whereas walking was more prevalent in the non-metropolitan region. Although all prevalences were lower in the 2 weeks prior to the survey than for the 12-month period, the patterns of variation according to gender, age and region were very similar (see Table 4).

The percentages of participants in each activity type who also participated in club sport (column d in Tables 3 and 4) provide a measure of the relative prevalence of sport participation among participants in each type of PA. Each percentage is either higher or lower than the corresponding proportion of club sport participants who also participate in the PA types (column e) according to whether the number of participants in the activity (column a) is lower or higher than the number of club sport participants (column b).

The gamma statistics in Tables 3 and 4 indicate that participation in weight training was significantly related to club sport participation in only three subsamples, although because the numbers involved were relatively small, the power to detect relationships was correspondingly low. The relationship was negative for 15–29 year olds and for non-metropolitan respondents (both 12-month and 2-week timeframes) and positive for metropolitan respondents (2-week timeframe only).

Participation in aerobics/fitness training was in general significantly related to club sport participation. The only two exceptions were 50+ age group (both 12-month and 2-week timeframes) and females (2-week timeframe only). All the significant relationships were negative, indicating lower proportions of club members than non-club members participating in this activity.

Participation in walking was in general significantly related to club sport participation. The only exception was the 50+ age group (2-week timeframe only). All the significant relationships were negative, indicating lower proportions of club members than non-club members participating in this activity.

Participation in running was in general significantly related to club sport participation. The exceptions were the 15–29 age group (both 12-month and 2-week timeframes), and males and non-metropolitan (2-week timeframe only). All the significant relationships were positive, indicating higher proportions of club members than non-club members participating in this activity.

The final two columns of Tables 3 and 4 further quantify the strength of these relationships. Column h shows the percentage of non-club sport participants who participated in the particular activity, and column i shows the difference between the percentages of club sport participants and non-club sport participants who participated in the particular activity. Overall, the proportion of club sport participants who participated in each of these four activities differed considerably from the proportion of non-club sport participants (Tables 3 and 4, column h). Compared to non-club-sport participants, in the past 12 months club sport participants were more likely to participate in running (difference 6.1 percentage points), but less likely to participate in walking (18.2 percentage points difference) or aerobics/fitness (3.9 percentage points difference). For weight training, the difference was negligible (0.2 percentage points). More details about differences for gender, age and area of residence can be found in Table 3 (previous 12 months) and Table 4 (previous 2 weeks).

## Discussion

Overall, club-based sport participation contributes considerably to LTPA. Club-based sport participation contributes to nearly a fifth of all HELPA LTPA (18 % of activities in the previous 12 months), and a third of all HELPA sport participation is conducted in a club-based setting. Furthermore most (78 %) of the HELPA sport participation was at a frequency of more than 12 times in the previous year.

These findings indicate that there is potential for sport to improve health through increased HELPA and for sport clubs to act as a setting to promote this. However, little attention has been focused on how sport can be managed as a means to promote active lifestyles or serve as a setting for interventions to promote healthy PA behaviour [17]. This is despite evidence of additional health benefits of participation in organised sport above and beyond physical health, whereby club sport participation by adults has been shown to have greater health benefits at low to moderate exposures than activities such as walking or working out at a gymnasium [6, 18]. Similarly, Vella and colleagues reported that participation in organised sport was associated with an increased likelihood of meeting PA guidelines in adolescence [19]. With regard to the health-related PA target of 10,000 steps per day, participation in organised PA has been shown to be associated with an increase in the number of steps taken daily compared to non-participation in organised PA [20].

Sporting organisations have been identified as a setting for promoting health through promotion of PA [21], particularly in Australia [22–26] and Scandinavia [27, 28]. Conversely, sporting organisations in Australia have been funded to implement health promotion policies and practices in order to create healthy sporting environments as a mechanism for broadening the appeal of sports clubs and thereby increasing community participation in sport [25]. Research is growing in this area—generating consensus on priority health promotion objectives for community sports clubs [29] and exploring the contribution of sport club participation to health-related quality of life [6]. The focus on sport in health promotion initiatives to promote PA, however, has been limited to facilitating changes in the sporting environment and has not focused on facilitating organisational changes, such as the way sport is organised and structured. This is despite evidence that the competitive nature and time demands of sport have been reported as factors influencing sport participation dropout. In particular, many adolescent girls perceive that club-based sport is so competitive that they cannot gain a position on a team [30]. Further, as adolescent girls age there is a tendency for their participation in PA to change from organised competitive activities to individual-based PA due to increasing demands of study and part-time or casual work [31]. Consideration of settings is a central feature of health promotion [25], and sports clubs have the potential to be an appropriate setting for health promotion programs and strategies to increase LTPA participation. However leisure settings such as sports clubs remain underutilised for health promotion [32].

It is acknowledged that while many health benefits are associated with participation in sport, some undesirable public health factors are also sometimes associated with club sport, including excessive alcohol consumption and smoking [33]. In light of this, leading health promotion agencies such as VicHealth in Australia are providing funding to develop and implement health promotion policies and practices within sports clubs [25, 34]. However, despite the potential of sports clubs for health promotion, there is research suggesting that sports clubs, being run largely by volunteers, and focused primarily on participation and competition, may not have the capacity to implement health promotion principles and policies [35] and may not accord high priority to health promotion [36] Furthermore, the predominantly volunteer nature of sports clubs can also limit their capacity to manage increases in participation [37].

The foundations of sport are deeply embedded within a traditional structure, with sport commonly organised around a competition fixture rather than opportunities for recreational participation. This is likely to be influenced by the fact that investment in sport has tended to prioritise elite performance rather than community participation [38]. In particular, many sports governing bodies are funded on the basis of their elite level performances and there are few incentives for directing resources to non-organised and/or recreational sport participation. The role and contribution of sport to PA promotion may be under-recognised for community wellbeing purposes [17, 39]. As such, building capacity to use sport as a setting for sustainable health promotion and specifically PA promotion has been identified as a challenge that will require continued investment and resources [21].

Turning to Tables 3 and 4, we focused initially on the second column of percentages (column e), because we postulated that these four types of PA are more likely to be undertaken in order to support participation in club sport than the reverse. The column e percentages provide an indication of the importance of club sport for promoting other forms of PA. To the extent that the participation in the ‘other’ activity is for the purpose of preparing and enhancing the capacity for the sporting activity, then these percentages would notionally represent an extra quantum of PA indirectly attributable at least in part to club sport, over and above the activity directly associated with club sport.

Of course, it is unlikely that all of this ‘other’ activity is driven entirely by sport participation. Furthermore, it is not possible to calculate from ERASS data a measure of the dosage or volume of activity (frequency × duration × intensity) associated with each particular reported activity, and so the percentages do not precisely represent a percentage increase in the dosage of PA. Notwithstanding these limitations, these percentages may provide a broad indication of the extent of a hidden benefit of sport in the form of preparatory or ancillary PA.

Turning to the first column of percentages in Tables 3 and 4 (column d), these may be regarded as providing a measure of the relative importance of sport participation in driving participation in each type of PA. This is most clearly demonstrated in the case of weight training. Only 2.8 % of club sport participants undertook weight training in the 12 months prior to the survey, but they represented 20.9 % of all those who undertook weight training. Conversely, 21.6 % of club sport participants walked in the 12 months prior to the survey, but they represented only 13.1 % of all those who walked.

However, to complete the picture, we must also consider participation in these four activities among non-sport club participants. When we do this, we see that overall, participation in aerobics/fitness training and walking were negatively associated with club sport participation. From this perspective, these activities may be seen as providing alternatives to club sport participation, rather than being promoted by club sport participation. Further, relatively small numbers participate in weight training, and there was little discernible evidence of any relationship with club sport participation. Only in the case of running was the proportion of participants higher among club sport participants than non-club sport participants, indicating a tendency for participation in club sport to result in extra running activity.

Limitations to this study include the lack of duration and intensity in ERASS data, and the fact that ERASS is based on retrospective self-report regarding periods of 2 weeks and 12 months duration. While the use of METs provided a proxy for intensity, the potential biases due to retrospective self-report were unavoidable.

## Conclusion

Overall, club-based sport participation contributes considerably to LTPA in general. Furthermore, nearly all sport participation is at a health enhancing level. Therefore, sports clubs play an important role as a setting for LTPA and more broadly promoting health through participation in sport. However, the structure of the provision of sport through clubs requires ongoing review, given the changing nature of the desires and preferences of actual and potential participants regarding structure and flexibility of LTPA pursuits.

Sport participants were significantly less likely than non-sport participants to participate in aerobics/fitness training and walking. This suggests that these non-sport activities are undertaken as alternatives to sport participation rather than as additional activities for the purpose of sport training. However sport participants were significantly more likely than non-sport participants to run, suggesting that running is used, in part at least, as a training activity for sport. This may be related to the higher intensity of running compared to walking and in many instances, to aerobics/fitness training also.

Health promotion policy, and more specifically PA policy, should give more consideration to the opportunities that sport can provide for health promotion. Furthermore, sport policy should recognise the role that sport plays for health in addition to the elite pathway focus. As to future research directions, it would be beneficial to investigate longitudinally the specific health benefits of participation in different types of sport, and to explore in more detail the dose—response relationship of sport participation (including frequency, duration and intensity) and health benefits, including the identification of thresholds.

## Abbreviations

ASC: Australian Sports Commission
ERASS: Exercise, Recreation and Sport Survey
HELPA: Health-enhancing leisure-time physical activity
LTPA: Leisure-time physical activity
NSO: National sporting organisation
PA: Physical activity
SSA: State sporting association
